# Comparative transcriptomics reveals the immune dynamics during the molting cycle of swimming crab *Portunus trituberculatus*

**DOI:** 10.3389/fimmu.2022.1037739

**Published:** 2022-10-31

**Authors:** Meimei Liu, Hongwei Ni, Xiaokang Zhang, Qiufeng Sun, Xugan Wu, Jie He

**Affiliations:** ^1^ Zhejiang Marine Fisheries Research Institute, Key Laboratory of Mariculture & Enhancement of Zhejiang Province, Zhoushan, China; ^2^ Jiangsu Key Laboratory of Marine Bioresources and Environment, Jiangsu Ocean University, Lianyungang, China; ^3^ Centre for Research on Environmental Ecology and Fish Nutrition of Ministry of Agriculture and Rural Affairs, Shanghai Ocean University, Shanghai, China

**Keywords:** *Portunus trituberculatus*, molting cycle, immunity, antioxidative enzyme system, comparative transcriptome

## Abstract

Molting is one of the most important biological processes of crustacean species, and a number of molecular mechanisms facilitate this complex procedure. However, the understanding of the immune mechanisms underlying crustacean molting cycle remains very limited. This study performed transcriptome sequencing in hemolymph and hepatopancreas of the swimming crab (*Portunus trituberculatus*) during the four molting stages: post-molt (AB), inter-molt (C), pre-molt (D), and ecdysis (E). The results showed that there were 78,572 unigenes that were obtained in the hemolymph and hepatopancreas of *P. trituberculatus*. Further analysis showed that 98 DEGs were involved in immunity response of hemolymph and hepatopancreas, and most of the DEGs participated in the process of signal transduction, pattern recognition proteins/receptors, and antioxidative enzymes system. Specifically, the key genes and pathway involved in signal transduction including the *GPCR126*, *beta-integrin*, *integrin*, three genes in mitogen-activated protein kinase (MAPK) signaling cascade (*MAPKKK10*, *MAPKK4*, and *p38 MAPK*), and four genes in Toll pathway (*Toll-like receptor*, *cactus*, *pelle-like kinase*, and *NFIL3*). For the pattern recognition proteins/receptors, the lowest expression level of 11 genes was found in the E stage, including *C-type lectin receptor*, *C-type lectin domain family 6 member A* and *SRB3/C* in the hemolymph, and hepatopancreatic *lectin 4*, *C-type lectin*, *SRB*, *Down syndrome cell adhesion molecule homolog*, *Down syndrome cell adhesion molecule isoform*, and *A2M*. Moreover, the expression level of *copper/zinc superoxide dismutase isoform 4*, *glutathione peroxidase*, *glutathione S-transferase*, *peroxiredoxin*, *peroxiredoxin 6*, and *dual oxidase 2* in stage C or stage D significantly higher than that of stage E or stage AB. These results fill in the gap of the continuous transcriptional changes that are evident during the molting cycle of crab and further provided valuable information for elucidating the molecular mechanisms of immune regulation during the molting cycle of crab.

## Introduction

Molting, a phenomenon of shedding the old exoskeleton and re-generating the new one, is one of the most important biological processes of crustacean species, and it is generally divided into three types: larval-metamorphic molts, growth-related molts, and reproductive molts (1, 2). The successful molting of crustacean will contribute to its growth, development, reproduction, and appendage regeneration (3–5). Consequently, the crabs and shrimp undergo dramatic biochemical and physiological changes during the molting stage. Previous studies showed that each molting cycle of crustacean can be normally divided into four recurrent stages, namely, inter-molt (stage C), pre-molt (stage D), ecdysis (stage E), and post-molt (stage AB), according to the morphological differences of epidermis, exoskeleton and the internal setal development of the appendages (6, 7). It has been well known that the crustaceans are too vulnerable to the attacks from other congeners or natural enemy during the period of molting or just finished molting, which leads to their death (8). It can be speculated that the changes in the immune response in crustaceans are closely related to the molting stage.

To date, several studies have shown that crustaceans exhibit different immune responses to pathogens at different molting stages, including white spot syndrome virus (WSSV) and pesticide virus (9, 10). Meanwhile, a few studies have analyzed immune dynamics during the molting cycle of crustaceans using transcriptomic techniques. For example, Xu et al. (11) reported that the expression level of genes related to antimicrobial peptide, Toll signaling pathway factors, phenoloxidase system, and antioxidant enzymes in the hepatopancreas of mud crab (*Scylla paramamosain*) were significantly upregulated at the post-molt stage and inter-molt stage compared with the pre-molt stage (11). Zhang et al. (12) founded that a significant positive correlation between the expression of two antimicrobial peptide (AMP) genes (SpALF5 and SpCrustin) and the relative abundance of two predominant microorganisms (Halomonas and Shewanella) in hemolymph was observed in the whole molt cycle of *S. paramamosain* (12). Gao et al. (6) reported that shrimp will upregulate the expression of the antimicrobial peptides in pre-molt stage for generate an immune response (6). Despite these previous findings, the understanding of the immune mechanisms underlying crustacean molting cycle remains very limited.

The swimming crab *Portunus trituberculatus* (Crustacea, Decapoda, and Brachyura) widely distributed along the coasts of East Asia countries and forms important fisheries in these countries (13–15). Due to its rapid growth rate and higher farming profit, the pond culture of *P. trituberculatus* has been developing rapidly in China (16, 17). The aquaculture production of *P. trituberculatus* reached up to 100,895 tonnes in 2020 in China (18). However, the survival rate of pond-reared *P. trituberculatus* is very low, only 5%, which is one of the important problems hindering the improvement of economic value and aquaculture profit for this species (8, 19). The reason for the low survival rate of pond-reared *P. trituberculatus* may be related to their higher cannibalism rate and various diseases during the molting period (8, 19, 20). Therefore, in order to sustain the stable swimming crab culture, it is very important to understand its immune response mechanism during molting cycle.

Similar to other crustaceans, the immune response of *P. trituberculatus* was also mediated by hemolymph and hepatopancreas. To further identify the molecular events altered during the four molting stages, post-molt, inter-molt, pre-molt, and ecdysis, herein, we used RNA-seq to investigate the immune response in the hemolymph and hepatopancreas of *P. trituberculatus* during the molting cycle. The associated findings in this study will provide an improved understanding of the molecular mechanism for the immune defense in crab and other molting animals.

## Materials and methods

### Ethics statement

All crabs were treated in strict accordance with the guidelines for the care and use of experimental animals established by the Administration of Affairs Concerning Experimental Animals of the State Council of the People’s Republic of China and approved by the Committee on Experimental Animal Management of the Jiangsu Ocean University.

### Experimental design and sampling

**Figure 1:**
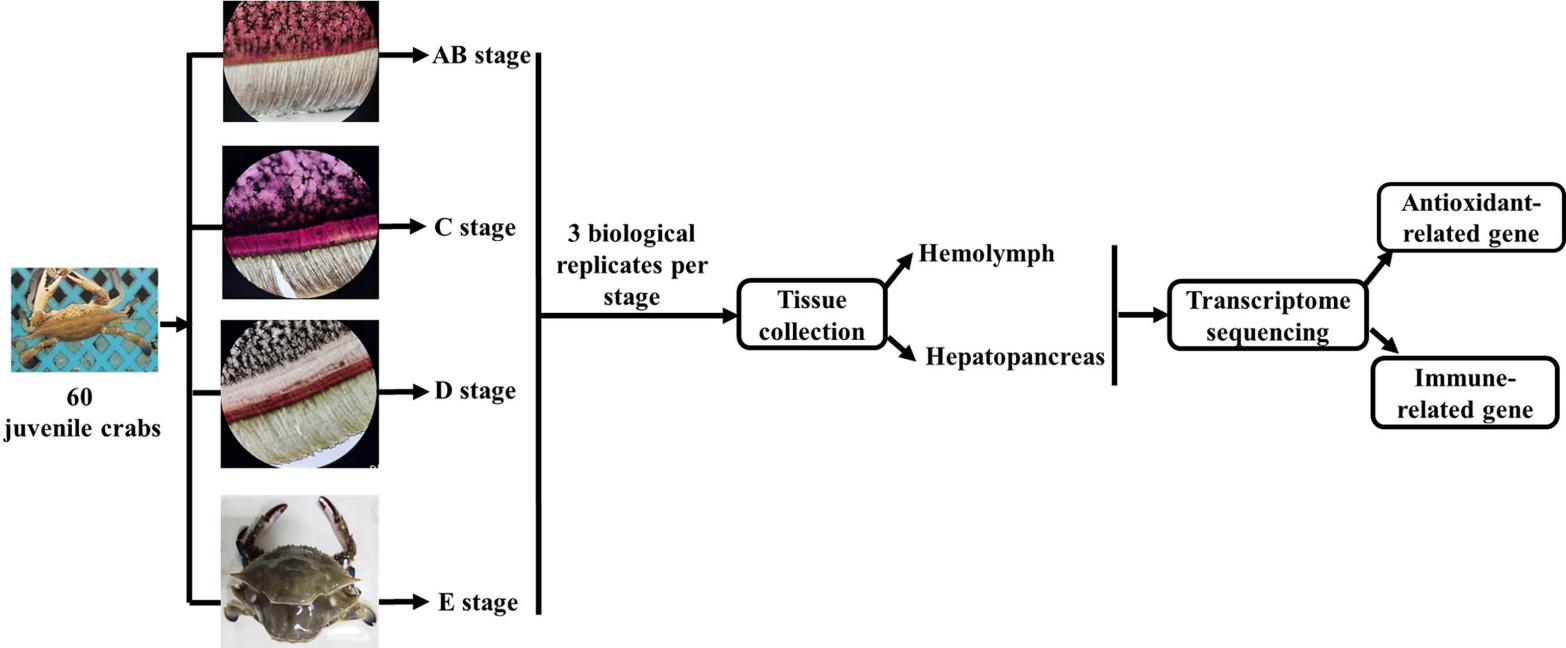
Schematic showing the experimental design and sampling procedures for the transcriptome analysis during the molting cycle of *P. trituberculatus*. Notes: The molting stage of *P. trituberculatus* was determined by observing the base of the swimming leg under a microscope before sampling. The characteristics of swimming leg bases in different molting stages of *P. trituberculatus* are shown in the figure. Stage AB: post-molt; stage C: inter-molt; stage D: per-molt; stage E: ecdysis.

The experimental design and sampling procedures are shown in **Figure 1**. Juvenile *P. trituberculatus* (body weight: 15–30 g) was collected from outdoor ponds of Qidong scientific research base of Shanghai Fisheries Research Institute, Jiangsu, China and then acclimated in an indoor circulating water system. During the acclimation, each crab was individually housed in culture baskets (Length × Width × Depth = 33 × 27.5 × 35 cm), and all baskets were kept floating in four concrete tanks (Length × Width × Depth = 5.8 m × 2.4 m × 1.8 m) supplied by filtered and treated recirculating seawater. After the crab finished molting, it was used for sampling of different molt cycles.

The molt cycle of *P. trituberculatus* was classified into four stages (post-molt [stage AB], inter-molt [stage C], per-molt [stage D], and ecdysis [stage E]) according to the previously report (21). Specifically, post-molt (AB) stage: *P. trituberculatus* has higher water content in its body, dorsal carapace edge hard, and the cuticle thickness is between 10 and 35 mm; inter-molt (C) stage: All parts of the body become hard, cuticle thickness is between 35 and 45 mm, and new carapace begins to secrete; per-molt (D) stage: All parts of the body is hard, the secretion of the outer epidermis is completed, and the new carapace is completely separated from the old carapace during dissection in later stage; ecdysis (E) stage: The new shell is clearly visible and the body can further absorb water. Three individual crabs at each stage were sampled, anesthetized on ice and then the hemolymph, and hepatopancreas were removed from the crabs for transcriptome analysis.

### RNA extraction, cDNA library preparation, and de novo assembly

Total RNA was isolated from each sample using the mirVana miRNA Isolation Kit (Ambion) according to manufacturer’s protocol. The purity and integrity of total RNA were estimated by a NanoDrop 2000 (Thermo Fisher Scientific, Massachusetts, USA) and 2100 Bioanalyzer (Agilent), respectively. Only high-quality RNA samples (OD260/OD280 ranged 1.8–2.2, RIN ≥ 8.0) were used to construct the sequencing library.

The libraries were constructed using TruSeq Stranded mRNA LTSample Prep Kit (Illumina) according to the manufacturer’s instructions. Then, these libraries were sequenced on the Illumina sequencing platform (HiSeqTM 2500 or Illumina HiSeq X Ten) and 150 bp paired-end reads were generated. The reads containing ploy-N and the low-quality reads were removed to obtain the clean reads. The clean reads were assembled into expressed sequence tag clusters (contigs) and *de novo* assembled into transcripts by using Trinityin paired-end method (22).

### Functional annotation and differential expression analysis

In order to obtain the annotation information of unigenes, the assembled unigenes were annotated with the NCBI non-redundant protein database (NR), Swiss-Prot protein and Clusters of orthologous groups for eukaryotic complete genomes (KOG) databases using BlastX with a threshold E-value of 1.0 × 10^−5^. The proteins with the highest hits to the unigenes were used to assign functional annotations thereto. Blast2go (http://www.BLAST2go.org/ ) and WEGO software were used to get the Gene Ontology (GO) annotation and GO functional classification for the unigenes. The unigenes were mapped to the Kyoto Encyclopedia of Genes and Genomes (KEGG) database to annotate their potential metabolic pathways.

The quantity expression of these unigenes was estimated by calculating FPKM (fragments per kb per million reads) using bowtie2 and eXpress to calculate FPKM. To identify differentially expressed genes (DEGs) across samples, |log_2_(fold change)| > 1 and *P*-value < 0.05 were set to be the thresholds for significantly different expression levels. The gene expression profiles were compared among the four stages and the DEGs were then subjected to enrichment analysis of GO functions and KEGG pathways.

### Expression levels of immune-related genes during the molting cycle using transcriptome data

To visualize the expression levels between different immune-related genes and molting stages, normalized counts in the form of transcripts per million values were generated. The expression level of immune-related genes was shown as log_2_ transcript per million plus one (TPM + 1) (23).

### Quantitative real-time PCR validation

Ten important genes involved in immune response during the molting cycle of *P. trituberculatus* were determined using qRT-PCR to validate the RNA-seq results. First-strand cDNA synthesis was performed with a reverse first strand cDNA synthesis kit (RR036A, Takara Bio, Japan). To normalize the target gene expression, 18*S* was used as an internal reference gene. Gene-specific primers were designed using Primer Premier 5.0 and listed in **Table 1**. The reaction system was performed as previously described (24). qRT-PCR was carried with a StepOnePlus™ real-time PCR system using SYBR1Green I (TakaRa, Japan) according to the manufacturer’s instructions. The relative expression level of each gene was calculated using the the 2^−ΔΔCt^ method (25). Data are presented as the mean ± standard error (SE).

**Table 1:** Specific primers used to in qRT-PCR.

Primer name	Sequence (5′→3′)
*GPCR126*	F: *TCTCTCTCCTGGGCTTCACAT* R: *AACCACTCCTTCCATAGTTTTCG*
*Integrin*	F: *CTTGGGTTTGGCTCTTTCGT* R: *TGGTGCCTCCTGGACCTTCT*
*MAPKK4*	F: *TTGATGAGAGAGAACAGAAGCAGC* R: *TTGTAGAATTTGTCCAGAGAGGTG*
*p38 MAPK*	F: *ATTCTGGACTTTGGATTGGCC* R: *TTGAGGTACTCCTGAGGGGG*
*Toll-like receptor*	F: *CTGAGAGTGGTAAGTGGCGG* R: *GAAGGAATGTGGACGGATCG*
*Cactus*	F: *AGGACTCACCCCATACCAGC* R: *CATCCATTATTCCCCAACCG*
*Pelle-like kinase*	F: *TCCTTCACCAGCCTCTCCGA* R: *ACCAAATCCCCCACGTCCTA*
*NFIL3*	F: *CCTCAGATGAGCGGGATTCT* R: *TGTTGCTGCTGGGGTGGATA*
*C-type lectin*	F: *CTTCACACACAACCAAGCGG* R: *AGCAGAACTAAGGGAGGCACA*
*Glutathione peroxidase 3*	F: *ATCCTAGCCTTCCCTTGCAA* R: *GAGCGGGTGTTCGTTCTTCC*
*18S*	F: *TCCAGTTCGCAGCTTCTTCTT* R: *AACATCTAAGGGCATCACAGACC*

## Results

### Transcriptome sequence assessment and gene functional annotation

After filtration, a total of 384.74 million reads were obtained from the four molting stages of *P. trituberculatus* transcriptomes, namely, 48.35 (AB-Ha), 47.98 (C-Ha), 48.85 (D-Ha), and 47.93 (E-Ha) million reads from the hemolymph libraries and 46.96 (AB-H), 49.01 (C-H), 48.38 (D-H), and 47.28 (E-H) million reads from the hepatopancreas libraries (**Table 2**). Finally, 78,572 unigenes were obtained after combining the transcripts, with an average length of 2654 bp, and the N50 was 4407 bp. Among the hemolymph unigenes, 38,570 (85.33%) unigenes could be matched to the NR database, 27 517 (60.88%) unigenes matched in the eggNOG database, whereas 10,978 (24.29%) unigenes matched in the KEGG (**Table 3**). For the hepatopancreas, 36,224 (86.62%) unigenes could be matched to the NR database, 26,834 (64.16%) unigenes were annotated in the eggNOG database, whereas 11,164 (26.69%) unigenes could be fully annotated with the KEGG (**Table 3**). The ILLUMINA data were submitted to the National Center for Biotechnology Information (NCBI, PRJNA855992).

**Table 2:** Raw reads and quality control of reads for cDNA libraries of *P. trituberculatus*.

Tissue	Stages	Number of raw reads (million reads)	GC (%)	Q30 (%)	Number of clean reads (million reads)
**Hemolymph**	Post-molt (AB)	49.46	48.68	93.27	48.35
	Inter-molt (C)	48.97	48.33	93.56	47.98
	Per-molt (D)	50.02	49.19	93.3	48.85
	Ecdysis (E)	49.22	46.33	92.95	47.93
**Hepatopancreas**	Post-molt (AB)	48.09	49.67	93.36	46.96
	Inter-molt (C)	49.99	50.15	93.87	49.01
	Per-molt (D)	49.33	49.67	93.84	48.38
	Ecdysis (E)	48.55	46.52	92.83	47.28

**Table 3:** Summary of functional annotation of *P. trituberculatus* transcriptome.

Tissue	Annotation in database	Unigene no.	Percentage (%)
**Hemolymph**	NR	38570	85.33
	Swissport	24649	54.53
	KEGG	10978	24.29
	KOG	22101	48.90
	EggNOG	27517	60.88
	GO	22890	50.64
	Pfam	24666	54.57
**Hepatopancreas**	NR	36224	86.62
	Swissport	24461	58.49
	KEGG	11164	26.69
	KOG	21974	52.54
	EggNOG	26834	64.16
	GO	22641	54.14
	Pfam	24895	59.53

### Analysis of differentially expressed genes (DEGs)

**Figure 2:**
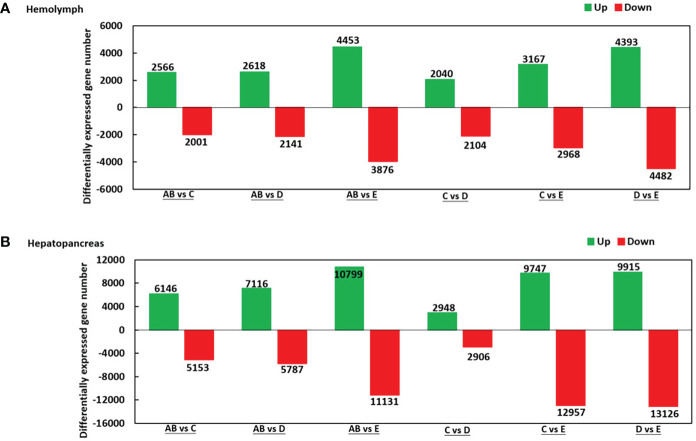
Summary of differentially expressed genes (DEGs) in transcriptome of hemolymph **(A)** and hepatopancreas **(B)** of *P. trituberculatus*. Notes (1): AB: post-molt; C: inter-molt; D: per-molt; E: ecdysis (2). Up- and downregulated genes are shown in green and red, respectively (3). The *x*-axis shows the comparisons between two stages, whereas the *y*-axis represents the total number of DEGs for each comparison. The numbers on the columns represent the number of DEGs.

As shown in **Figure 2**, there were 2.566 upregulated genes and 2,001 downregulated genes in AB-Ha *versus* C-Ha, 2,618 upregulated genes and 2,141 genes downregulated in AB-Ha *versus* D-Ha, 4,453 upregulated genes and 3,876 genes downregulated in AB-Ha *versus* E-Ha, 2,040 upregulated genes and 2 104 genes downregulated in C-Ha *versus* D-Ha, 3,167 upregulated genes and 2,968 genes downregulated in C-Ha *versus* E-Ha, 4,393 upregulated genes and 4,482 genes downregulated in D-Ha *versus* E-Ha. For the hepatopancreas, 6,146 upregulated genes and 5,153 downregulated genes in AB-H *versus* C-H, 7,116 upregulated genes and 5,787 genes downregulated in AB-H *versus* D-H, 10,799 upregulated genes and 11,131genes downregulated in AB-H *versus* E-H, 2,948 upregulated genes and 2,906 genes downregulated in C-H *versus* D-H, 9,747 upregulated genes and 12,957 genes downregulated in C-H *versus* E-H, 9,915 upregulated genes and 13,126 genes downregulated in D-H *versus* E-H (**Figure 2**).

**Figure 3:**
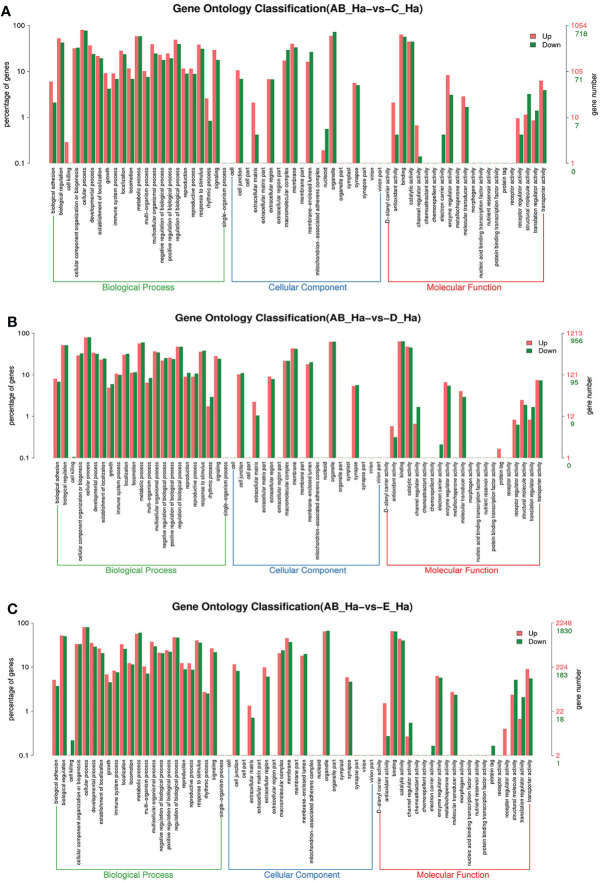
GO enrichment analyses of differentially expressed genes (DEGs) in hemolymph transcriptome. Notes: **(A)** Comparison between AB-Ha and C-Ha; **(B)** comparison between AB-Ha and D-Ha. **(C)** Comparison between AB-Ha and E-Ha. Notes (1): AB-Ha: hemolymph from post-molt; C-Ha: hemolymph from inter-molt; D-Ha: hemolymph from per-molt; E-Ha: hemolymph from ecdysis (2). Red columns indicate GO item entries enriched by upregulated differentially expressed genes; green columns indicate GO item entries enriched by downregulated differentially expressed genes. (3) The *x*-axis shows the item name between two stages, whereas the *y*-axis represents the number of genes and their percentage of the corresponding item.

**Figure 4:**
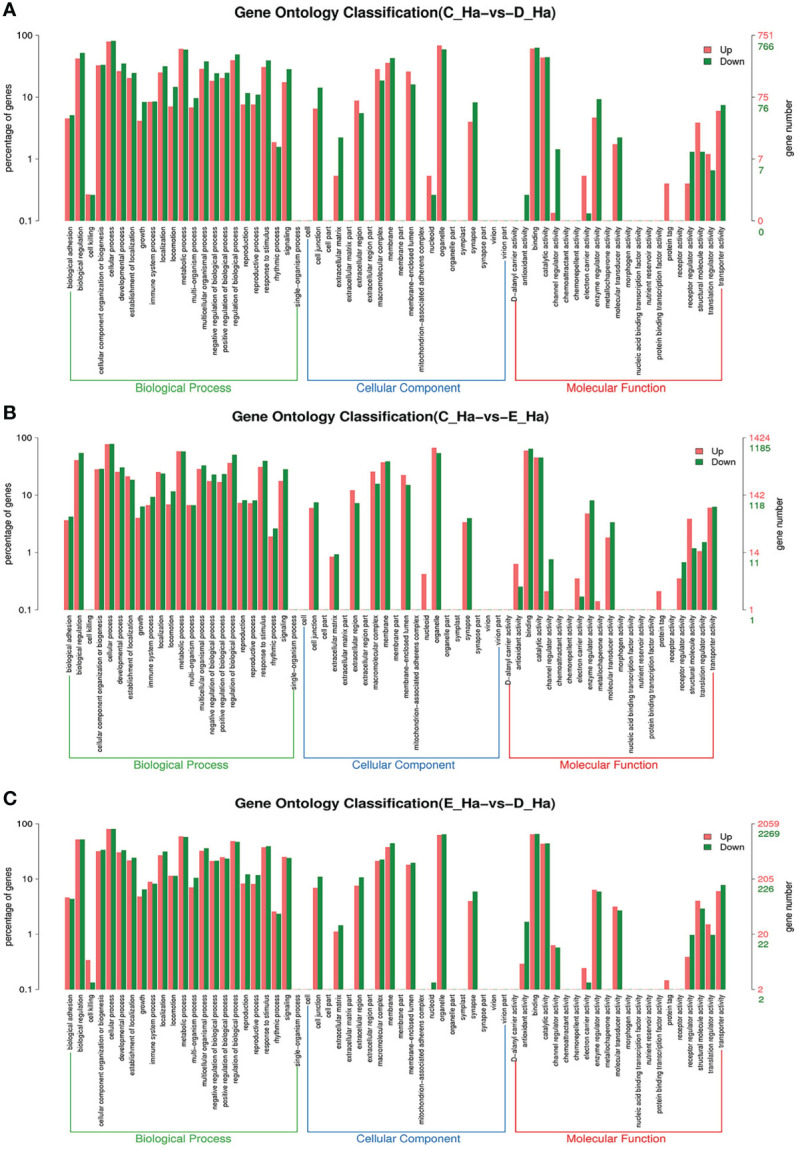
GO enrichment analyses of differentially expressed genes (DEGs) in hemolymph transcriptome. Notes: **(A)** Comparison between C-Ha and D-Ha; **(B)** comparison between C-Ha and E-Ha. **(C)** Comparison between E-Ha and D-Ha. Notes: (1) C-Ha: hemolymph from inter-molt; D-Ha: hemolymph from per-molt; E-Ha: hemolymph from ecdysis. (2) Red columns indicate GO item entries enriched by upregulated differentially expressed genes; green columns indicate GO item entries enriched by downregulated differentially expressed genes (3). The *x*-axis shows the item name between two stages, whereas the *y*-axis represents the number of genes and their percentage of the corresponding item.

**Figure 5:**
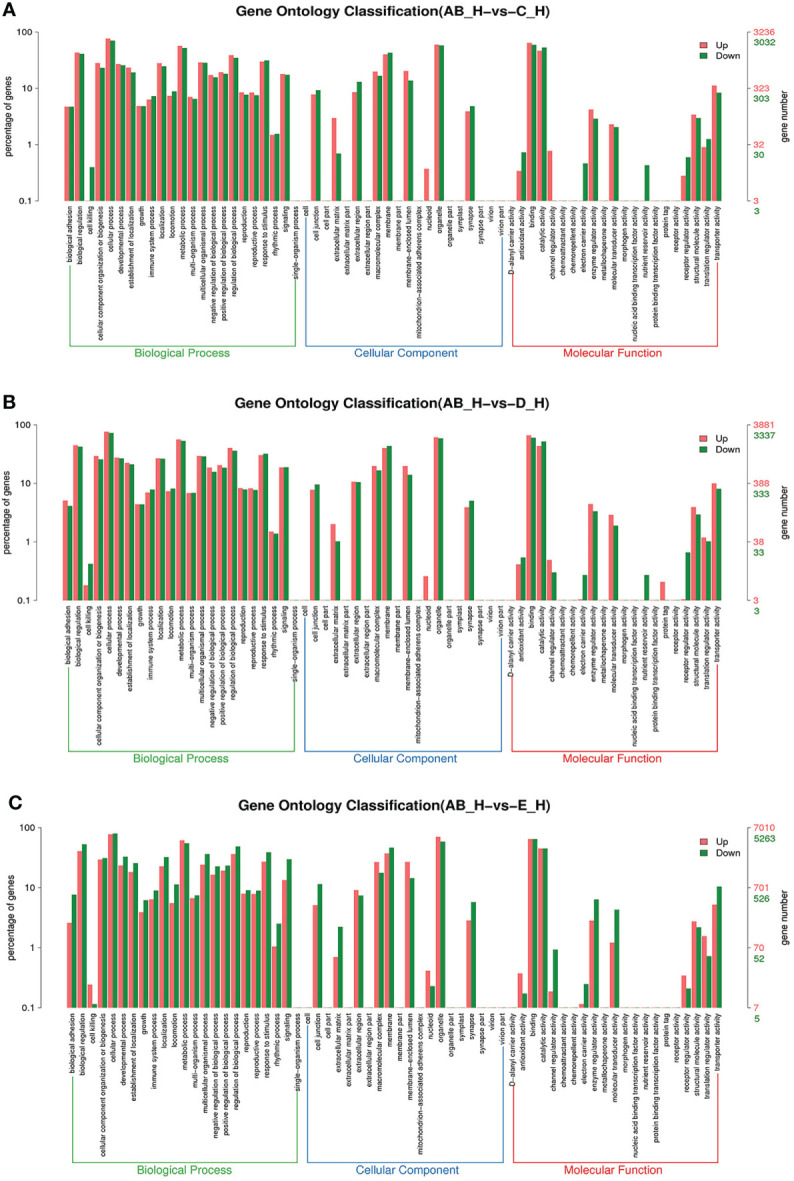
GO enrichment analyses of differentially expressed genes (DEGs) in hepatopancreas transcriptome. Notes: **(A)** Comparison between AB-H and C-H; **(B)** comparison between AB-H and D-H. **(C)** Comparison between AB-H and E-H. Notes: (1) AB-H: hepatopancreas from post-molt; C-H: hepatopancreas from inter-molt; D-H: hepatopancreas from per-molt; E-H: hepatopancreas from ecdysis. (2) Red columns indicate GO item entries enriched by upregulated differentially expressed genes; green columns indicate GO item entries enriched by downregulated differentially expressed genes. (3) The *x*-axis shows the item name between two stages, whereas the *y*-axis represents the number of genes and their percentage of the corresponding item.

**Figure 6:**
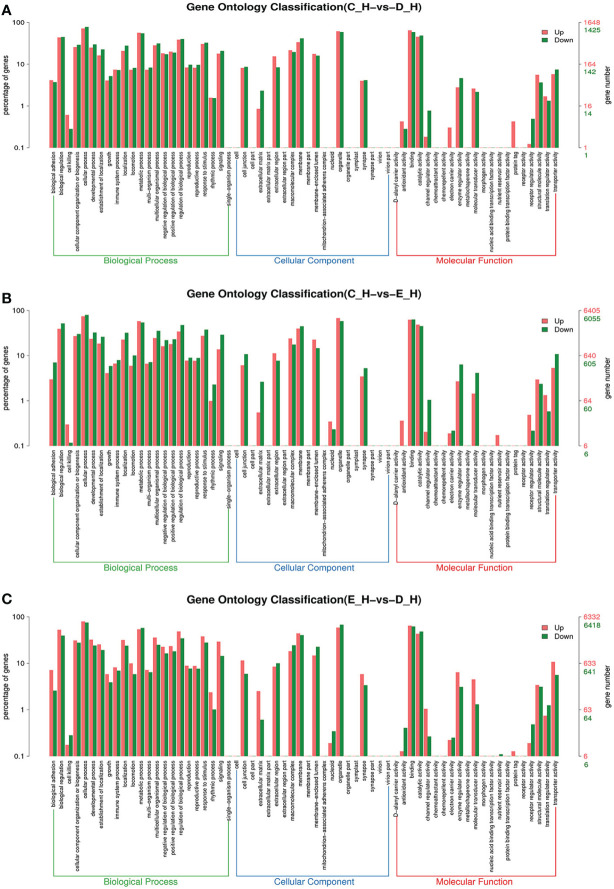
GO enrichment analyses of differentially expressed genes (DEGs) in hepatopancreas transcriptome. Notes: **(A)** Comparison between C-H and D-H; **(B)** comparison between C-H and E-H. **(C)** Comparison between E-H and D-H. Notes: (1) C-H: hepatopancreas from inter-molt; D-H: hepatopancreas from per-molt; E-H: hepatopancreas from ecdysis. (2) Red columns indicate GO item entries enriched by upregulated differentially expressed genes, green columns indicate GO item entries enriched by downregulated differentially expressed genes (3) The *x*-axis shows the item name between two stages, whereas the *y*-axis represents the number of genes and their percentage of the corresponding item.

To further assign the putative functions to DEGs, GO and KEGG analyses were performed. GO annotation output showed that the DEGs were mainly classified into three parts: biological process, cellular component, and molecular function. GO annotation of the DEGs in the different molting stage in the hemolymph showed that the DEGs were categorized mainly into cellular process (GO:0009987) and metabolic process (GO:0008152) in biological processes, organelle (GO:0031090) and membrane (GO:0016020) in cellular component, and catalytic activity (GO:0003824) and binding (GO:0005488) in molecular function, respectively (**Figures 3**, **4**). In the hepatopancreas, compared AB stage and E stage, a total of 6,268 DEGs were annotated with GO terms, 7,218 DEGs were annotated with GO terms between the AB stage and D stage, and 12,273 DEGs were annotated with GO terms between the AB stage and E stage. In the comparison of C stage and D stage, 3,073 DEGs were annotated with GO terms. When C stage and E stage were compared, 12,460 DEGs were assigned to 7,571 GO terms (level 2). In the comparison of D stage and E stage, 12,750 DEGs were annotated with GO terms. Overall, the GO assignment result of hepatopancreatic DEGs was similar to the hemolymph (**Figures 5**, **6**). KEGG enrichment results showed that the DEGs in the hemolymph were enriched in 331 specific KEGG metabolic pathways, whereas the DEGs in the hepatopancreas were enriched in 337 specific KEGG metabolic pathways. The top 10 most significantly enriched metabolic pathways in the hemolymph and hepatopancreas are represented in **Table 4**. In the hemolymph, the significantly enriched pathways included “maturity onset diabetes of the young,” “Jak-STAT signaling pathway,” “Bacterial invasion of epithelial cells.” The DEGs in the hepatopancreas were significantly clustered in “ribosome,” “amino sugar and nucleotide sugar metabolism,” and “collecting duct acid secretion.”

**Table 4:** Significantly changed pathways obtained with KEGG, using the significantly changed genes from hemolymph and hepatopancreas during the molt cycle of *P. trituberculatus*.

Tissue	Pathway	ID	DEGs number					
			AB *vs.* C	AB *vs.* D	AB *vs.* E	C *vs.* D	C *vs.* E	E *vs.* D
**Hemolymph**
	Maturity onset diabetes of the young	ko04950	13	7	17	-	16	17
	Jak-STAT signaling pathway	ko04630	-	-	28	11	20	39
	Bacterial invasion of epithelial cells	ko05100	-	44	-	-	-	-
	Ribosome	ko03010	-	-	77	25	-	64
	IL-17 signaling pathway	ko04657	17	-	28	-	-	36
	Ribosome biogenesis in eukaryotes	ko03008	117	111	-	96	129	-
	Leukocyte transendothelial migration	ko04670	-	36	-	-	-	-
	Ovarian steroidogenesis	ko04913	23	-	-	-	-	-
	Focal adhesion	ko04510	-	58	-	-	-	-
	Yersinia infection	ko05135	-	57	-	-	-	-
**Hepatopancreas**
	Ribosome	ko03010	-	-	-	83	-	-
	Amino sugar and nucleotide sugar metabolism	ko00520	-	-	137	37	156	170
	Collecting duct acid secretion	ko04966	34	-	61	-	50	55
	Pyruvate metabolism	ko00620	58	54	-	-	76	79
	AMPK signaling pathway	ko04152	-	-	-	-	167	182
	Vibrio cholerae infection	ko05110	-	-	117	-	-	-
	Protein digestion and absorption	ko04974	82	-	-	36	111	-
	Glycolysis/Gluconeogenesis	ko00010	75	-	-	27	109	107
	Aminoacyl-tRNA biosynthesis	ko00970	-	-	68	-	-	63
	Adipocytokine signaling pathway	ko04920	-	-	-	-	106	101

AB, post-molt; C, inter-molt; D, per-molt; E, ecdysis.

### Key genes involved in immunity response

According to the gene data, 98 DEGs are involved in various processes of crab immunity response in the hemolymph and hepatopancreas and those DEGs were categorized into nine groups (**Table 5**). Specifically, 25 key genes were mainly involved in signal transduction, namely, *G-protein coupled receptor 126* (*GPCR126*), *G-protein coupled receptor Mth2*, *mitogen-activated protein kinase kinase 4* (*MAPKK4*), *MAPKKK10*, *MAPKKK3, MAPKKK12, p38 MAPK*, *beta-integrin*, *integrin*, *protein kinase C and casein kinase substrate in neurons protein 1-like* and four genes (*Toll-like receptor*, *cactus*, *pelle-like kinase*, *nuclear factor interleukin 3-regulated transcription factor* [*NFIL3*]) in Toll pathway. Sixteen genes related to pattern recognition proteins/receptors were identified in the hemolymph and hepatopancreas, such as *lectin 4*, *C-type lectin*, *C-type lectin receptor*, *C-type lectin domain family 6 member A*, *C-type lectin-like domain-containing protein PtLP*, *scavenger receptor B* (*SRB*), *SRB3*, *SRC*, *Down syndrome cell adhesion molecule homolog*, *Down syndrome cell adhesion molecule isoform*, *alpha-2 macroglobulin* (*A2M*), *tetraspanin*, *tetraspanin-11*, *tetraspanin-2A-like*, *tetraspanin-13-like*, and *lipopolysaccharide and beta-1,3-glucan binding protein* (*LBGBP*). Ten genes related to other immune molecules were identified in the hemolymph and hepatopancreas, such as *metallothionein*, *metallothionein 2*, *macrophage migration inhibitory factor*, *macrophage migration inhibitory factor MIF1*, *macrophage migration inhibitory factor MIF2*, *hemocyanin*, *hemocyanin subunit*, *hemocyanin subunit 1*, *hemocyanin subunit 2*, and *hemocyanin subunit 5*. Nine genes related to apoptosis were identified in the hemolymph and hepatopancreas, such as *caspase*, *caspase 2*, *caspase 3*, *caspase 4*, *caspase 8*, *cathepsin A*, *cathepsin C*, *neprilysin-11*, and *autophagy protein 5*. For the prophenoloxidase cascade, seven genes were identified in the hemolymph and hepatopancreas, namely, *prophenoloxidase*, *prophenoloxidase activating factor 1, serine proteinase, serine protease 4, serine protease 5, serine protease 27*, and *serine proteinase inhibitor 2.* For the antimicrobial peptides, six genes were identified in the hemolymph and hepatopancreas, namely, *lysozyme*, *crustin antimicrobial peptide*, *antimicrobial peptide hyastatin*, *anti-lipopolysaccharide factor isoform 4* (*ALF4*), *ALF5*, and *ALF7*. For the blood clotting system, the *transglutaminase and transglutaminase 2* were found in the hemolymph and hepatopancreas.

**Table 5:** Summary of differential expressed genes related to immune in the transcriptome of *P. trituberculatus*.

Functional category	Gene id	Full name	Tissue	FC^a^					
				AB *vs.* C	AB *vs.* D	AB *vs.* E	C *vs.* D	C *vs.* E	E *vs.* D
**Antimicrobial peptides**									
	MSTRG.10366.3	*Lysozyme*	Ha	-3.00^*^	0.51	2.18*	11.38	5.02*	1.97
	MSTRG.10366.3	*Lysozyme*	H	0.35	1.10*	-1.17*	0.73	-1.67*	2.43*
	MSTRG.27414.1	*Crustin antimicrobial peptide*	Ha	-6.53*	-4.00*	4.34*	2.52*	10.89*	-8.36*
	MSTRG.27414.1	*Crustin antimicrobial peptide*	H	-3.31*	-2.95*	1.93	0.34	5.10*	-4.77*
	MSTRG.15908.3	*Antimicrobial peptide hyastatin*	Ha	-1.98	-1.64*	-1.06	0.33	0.93	-0.59
	MSTRG.15908.3	*Antimicrobial peptide hyastatin*	H	-0.45	-0.43	0.74	-0.001	1.04*	-1.03*
	MSTRG.13545.2	*Anti-lipopolysaccharide factor isoform 5*	Ha	-2.24	-0.83	-2.28*	1.40	-0.05	1.44
	MSTRG.13545.2	*Anti-lipopolysaccharide factor isoform 5*	H	4.45*	-0.55	0.25	-5.02*	-4.33*	-0.65
	MSTRG.13550.4	*Anti-lipopolysaccharide factor isoform 4*	H	-2.71*	1.34	1.69	4.03*	4.26*	-0.12
	MSTRG.13550.4	*Anti-lipopolysaccharide factor isoform 4*	Ha	2.94	3.71*	1.72	0.79	-1.3	1.98
	MSTRG.22109.1	*Anti-lipopolysaccharide factor isoform 7*	Ha	-1.97	0.32	0.88	2.28	2.85*	-0.56
**Blood clotting system**									
	MSTRG.3725.10	*Transglutaminase 2*	Ha	7.98*	1.43	5.69*	-6.56*	-2.15	-4.27*
	MSTRG.3725.10	*Transglutaminase 2*	H	-0.61	7.84*	7.07*	8.43*	7.52*	0.93
	MSTRG.3063.6	*Transglutaminase*	Ha	1.11	-3.02*	0.88	-4.13*	3.64*	-3.93*
	MSTRG.3063.6	*Transglutaminase*	H	-1.44*	-2.65*	0.27	-1.63*	2.68*	-2.79*
**Prophenoloxidase cascade**									
	MSTRG.22599.2	*Serine proteinase*	Ha	0.40	1.76*	0.99	1.35	0.61	0.76
	MSTRG.22599.2	*Serine proteinase*	H	-0.49	4.36	1.91	2.60*	2.27	0.32
	MSTRG.10394.12	*Serine protease 4*	Ha	-1.57	-0.80	2.86*	0.77	4.44*	-3.67*
	MSTRG.10394.12	*Serine protease 4*	H	-3.15*	-3.96*	-3.12*	1.98*	-0.11	-0.69
	MSTRG.25062.13	*Serine protease 5*	Ha	-4.57*	-3.11	2.74	1.45	7.38*	-5.93*
	MSTRG.11992.1	*Serine protease 27*	Ha	-1.03	-0.03	-10.2*	1.00	-9.43*	10.2*
	MSTRG.9821.17	*Serine proteinase inhibitor 2*	Ha	4.84*	2.61*	9.28*	-3.72*	10.6*	-9.50*
	MSTRG.24061.1	*Prophenoloxidase*	Ha	1.60	-0.24	-3.94*	-1.84	-5.38*	3.67*
	MSTRG.24061.1	*Prophenoloxidase*	H	0.52	0.55	-1.14*	0.004	-1.82*	1.83*
	MSTRG.27447.1	*Prophenoloxidase activating factor 1*	H	0.27	1.71*	3.16*	1.42	-0.54	1.95*
**Pattern recognition proteins/receptors**									
	MSTRG.10002.1	*Lectin 4*	H	0.19	0.29	1.12*	0.07	0.78*	-0.69
	MSTRG.10849.1	*C-type lectin*	H	1.58*	2.88*	4.60*	1.27*	2.86*	-1.56*
	MSTRG.14768.3	*C-type lectin receptor*	Ha	-8.78*	-3.45	2.97	5.34*	8.59*	-3.29
	MSTRG.14768.3	*C-type lectin receptor*	H	10.3*	-5.09*	-2.21*	-15.4*	-12.7*	-2.73*
	MSTRG.24141.2	*C-type lectin domain family 6 member A*	Ha	-2.44	5.26*	6.46*	4.12	5.37	-1.27
	MSTRG.24141.2	*C-type lectin domain family 6 member A*	H	1.60*	2.54*	-1.32*	5.23*	-2.27*	7.53*
	MSTRG.25714.1	*C-type lectin-like domain-containing protein PtLP*	H	4.63*	2.09*	-1.75*	-2.56*	-6.52*	4.00*
	MSTRG.12523.17	*Scavenger receptor C*	Ha	2.49	5.73*	8.59*	3.23	4.24*	-2.87
	MSTRG.422.14	*Scavenger receptor B3*	Ha	1.38	-0.95	1.78	-2.35	0.41	-2.75*
	MSTRG.422.14	*Scavenger receptor B3*	H	1.06*	2.25*	0.03	1.17*	-1.18*	2.37*
	MSTRG.10379.18	*Scavenger receptor B*	H	-4.24*	-4.88*	6.97*	-1.68*	7.29*	-7.94*
	MSTRG.28302.2	*Down syndrome cell adhesion molecule homolog*	H	-3.74*	-0.93	1.92	2.78*	5.51*	-2.72*
	MSTRG.6614.2	*Down syndrome cell adhesion molecule isoform*	H	-3.68*	-3.48*	3.34	0.18	6.86*	-6.66*
	MSTRG.18936.1	*Lipopolysaccharide and beta-1,3-glucan binding protein*	H	-3.40*	-4.35*	-2.15	-0.97	1.10	-2.06
	MSTRG.17780.1	*Alpha-2 macroglobulin*	Ha	4.57	4.25*	1.97	-0.46	-2.70	2.26
	MSTRG.17780.1	*Alpha-2 macroglobulin*	H	-1.38*	0.39	1.12*	1.75*	2.44*	-1.66*
	MSTRG.6068.1	*Tetraspanin*	H	-1.51*	-1.15*	-1.86*	0.34	-0.50	0.86*
	MSTRG.22077.11	*Tetraspanin-11*	Ha	5.08*	0.07	3.45*	-5.02*	-1.59	-3.41*
	MSTRG.13619.2	*Tetraspanin-13-like*	H	1.94*	1.78*	-1.23*	-0.18	-3.32*	3.16*
	MSTRG.3525.1	*Tetraspanin-2A-like*	H	-1.34*	-1.64*	-1.83*	-0.32	-0.64	0.32
**Signal transduction**									
	MSTRG.14568.1	*G-protein coupled receptor 126*	Ha	-0.67	-0.91	1.99*	-0.25	2.70*	-2.93*
	MSTRG.3369.2	*G-protein coupled receptor 161*	H	-4.14*	-0.16	2.37	3.94*	6.35*	-2.37
	MSTRG.3198.2	*G-protein coupled receptor 101*	H	-2.02*	-1.87*	4.62*	-3.07*	4.12*	-3.95*
	MSTRG.11169.1	*G-protein coupled receptor Mth2*	Ha	6.43*	4.97*	2.67*	-1.57	-3.81	2.30
	MSTRG.5582.1	*G-protein coupled receptor Mth-like 3*	H	1.74*	1.61*	-1.38*	-0.15	-3.26*	3.13*
	MSTRG.13926.1	*G-protein coupled receptor Mth-like 10*	H	-1.79*	-1.51*	3.81*	0.27	5.46*	-5.18*
	MSTRG.27616.3	*Mitogen-activated protein kinase p38*	Ha	2.89	1.14*	-1.23	-2.73*	-4.11*	2.35*
	MSTRG.18056.12	*Mitogen-activated protein kinase kinase 4*	Ha	2.72	-1.73*	-2.32	-2.49	-4.99*	2.52*
	MSTRG.18056.12	*Mitogen-activated protein kinase kinase 4*	H	-1.95*	-2.01*	-3.22*	-0.07	-5.61*	2.69*
	MSTRG.4111.7	*Mitogen-activated protein kinase kinase kinase 10-like*	Ha	7.47*	1.44	-0.18	-6.04	-7.67*	1.61
	MSTRG.4111.7	*Mitogen-activated protein kinase kinase kinase 10-like*	H	1.32*	1.35*	-2.22*	0.005	-3.12*	3.15*
	MSTRG.7461.3	*Mitogen-activated protein kinase kinase kinase kinase 3-like*	H	5.37*	1.44	-2.17*	-3.93	-7.68*	3.74*
	MSTRG.20627.8	*Mitogen-activated protein kinase kinase kinase 12-like isoform X2*	H	1.42*	4.70*	-2.08*	3.27*	-2.14*	3.35*
	MSTRG.13086.9	*Protein kinase C and casein kinase substrate in neurons protein 1-like*	Ha	-0.27	1.59*	-0.69	1.86	-0.42	2.27*
	MSTRG.13086.9	*Protein kinase C and casein kinase substrate in neurons protein 1-like*	H	-0.12	1.23*	-1.28*	1.31*	-1.31*	2.65*
	MSTRG.24188.7	*Protein kinase C delta type homolog isoform X2*	H	1.14	0.99	-1.27*	-0.16	-2.55*	2.40*
	MSTRG.3383.6	*Integrin*	Ha	6.54*	1.24*	4.85*	-5.31*	-1.73	-3.63*
	MSTRG.3383.6	*Integrin*	H	1.62*	-2.44*	-2.44*	-1.92*	-3.48*	-0.06
	MSTRG.14654.11	*Beta-integrin*	Ha	-8.87*	-8.48*	-6.43*	0.38	2.42	-2.08
	MSTRG.3440.2	*Integrin beta 3*	H	-1.43*	-1.19*	-1.75*	0.22	-1.31*	1.31*
	MSTRG.15764.1	*Integrin alpha 4*	H	-1.57*	-1.45*	1.74*	0.10	3.16*	-3.04*
	MSTRG.2144.18	*Integrin alpha 5*	Ha	-0.04	4.79*	3.62*	4.83*	3.65*	-1.37*
	MSTRG.14056.10	*Integrin alpha-PS3-like*	H	1.24*	1.89*	-1.31*	2.35*	-2.70*	2.08*
	MSTRG.15681.1	*Toll-like receptor*	H	-0.97*	-0.87*	0.93*	0.09	1.75*	-1.65*
	MSTRG.1518.11	*Cactus*	Ha	-0.52	-0.28	4.22*	0.23	4.76*	-4.53*
	MSTRG.1518.11	*Cactus*	H	1.89*	2.58*	1.09	0.67	-0.59	1.27
	MSTRG.18683.1	*Pelle-like kinase*	H	-1.20*	-0.58	1.92	0.60	2.96*	-2.33
	MSTRG.12972.1	*Interleukin-1 receptor-associated kinase 4*	H	1.20*	2.58*	2.40*	-0.82	4.17*	-1.84*
	MSTRG.3449.32	*Interleukin enhancer-binding factor 2*	H	1.14*	1.38*	4.89*	0.21	3.58*	-3.35*
	MSTRG.25601.3	*Nuclear factor interleukin 3-regulated transcription factor*	Ha	2.84	2.87*	5.36*	0.03	2.54	-2.29*
**Apoptosis**									
	MSTRG.28846.7	*Caspase*	Ha	5.89*	5.74*	2.31*	-2.87*	0.57	-3.44*
	MSTRG.28846.7	*Caspase*	H	-1.10*	-1.01*	1.08*	0.07	2.04*	-1.95*
	MSTRG.3668.7	*Caspase 2*	Ha	-7.67*	-3.78	-8.96*	3.89	-1.27	5.18*
	MSTRG.8546.21	*Caspase 3*	Ha	6.36*	6.22*	-0.19	-0.30	-6.50*	6.38*
	MSTRG.8546.21	*Caspase 3*	H	-1.68*	1.73*	4.93*	1.66	6.47*	-4.85*
	MSTRG.23469.7	*Caspase 4*	H	1.25*	0.39	-1.69*	-0.88	-2.02*	1.15*
	MSTRG.24772.6	*Caspase 8*	H	-6.53*	-2.86	3.27*	3.65	3.11*	-2.67*
	MSTRG.11152.1	*Cathepsin A*	H	-1.10*	-0.98*	-0.98*	0.10	1.94*	-1.83*
	MSTRG.16241.4	*Cathepsin C*	H	-4.44*	-7.76*	-1.13	-3.34*	3.17*	-6.49*
	MSTRG.10422.6	*Neprilysin-11*	H	1.37*	2.12*	5.51*	0.73	3.98*	-3.24*
	MSTRG.28867.3	*Autophagy protein 5*	H	-0.14	0.63	1.10*	0.76	1.48*	-0.31
**Other immune molecules**									
	MSTRG.26983.1	*Metallothionein*	Ha	-3.59*	-1.25*	0.64	2.33	4.23*	-1.90*
	MSTRG.26983.1	*Metallothionein*	H	-0.65	-0.94	2.00*	-0.32	2.50*	-2.80*
	MSTRG.26991.33	*Metallothionein 2*	H	-2.93*	-4.77*	-3.26*	-1.86	-0.48	-1.35
	MSTRG.13713.1	*Macrophage migration inhibitory factor MIF2*	Ha	-1.98	-0.53	1.07	1.44	3.06*	-1.62*
	MSTRG.16594.6	*Macrophage migration inhibitory factor MIF1*	H	-1.50*	-0.99*	2.41*	0.48	3.75*	-3.24*
	MSTRG.20119.1	*Macrophage migration inhibitory factor*	H	-1.49	-1.43*	1.70	0.04	3.04*	-2.97*
	MSTRG.14472.1	*Hemocyanin*	H	-5.79*	-3.61*	3.86*	2.16*	9.49*	-7.30*
	MSTRG.26430.1	*Hemocyanin subunit*	H	-7.15*	-5.75*	2.58*	1.38*	9.58*	-8.17*
	MSTRG.26431.1	*Hemocyanin subunit 1*	Ha	-3.67*	-1.89	5.07	1.77	8.73*	-6.95*
	MSTRG.26431.1	*Hemocyanin subunit 1*	H	-5.97*	-3.65*	4.03*	2.30*	9.84*	-7.52*
	MSTRG.26434.1	*Hemocyanin subunit 2*	H	-5.78*	-3.99*	4.38*	1.77*	10.0*	-8.21*
	MSTRG.26427.9	*Hemocyanin subunit 5*	H	-4.19*	-3.66*	3.04	0.51	7.05*	-6.53*
**Antioxidative enzymes system**									
	MSTRG.25042.1	*NADPH oxidase*	Ha	-0.19	-0.65	-2.07*	-0.47	-1.87	1.39*
	MSTRG.25042.1	*NADPH oxidase*	H	-1.70*	-2.57*	-2.22*	-0.88	-0.65	-0.22
	MSTRG.10531.2	*Copper/zinc superoxide dismutase 2*	Ha	8.21*	2.59*	-0.97	-5.64*	-9.19*	3.54*
	MSTRG.14683.2	*Copper/zinc superoxide dismutase isoform 4*	H	-4.82*	-5.64*	-0.60	-0.84	4.16*	-4.97*
	MSTRG.10673.2	*Extracellular copper-zinc superoxide dismutase*	H	6.18*	3.43*	-1.63*	-2.77	-3.52*	0.77
	MSTRG.14542.4	*Glutathione peroxidase-like isoform X1*	Ha	-4.86*	-3.16*	-3.62*	1.69	1.24	0.47
	MSTRG.14542.4	*Glutathione peroxidase-like isoform X1*	H	-1.82*	-2.57*	-2.62*	-0.77	-0.95	0.20
	MSTRG.11789.3	*Glutathione peroxidase 7-like*	Ha	6.62*	0.84	2.54	-5.79	-4.06	-1.71
	MSTRG.11789.3	*Glutathione peroxidase 7-like*	H	-2.56*	-3.68*	-1.66	-1.13	0.74	-1.84
	MSTRG.2312.21	*Glutathione peroxidase*	H	-2.34*	-0.45	2.85*	1.87*	5.03*	-3.16*
	MSTRG.13899.1	*Glutathione peroxidase 3*	H	1.33*	1.72*	0.36	0.37	-1.13*	1.53*
	MSTRG.11980.1	*Glutathione S-transferase*	Ha	-2.67*	-1.00	-1.25	1.66	1.42	0.24
	MSTRG.11980.1	*Glutathione S-transferase*	H	-2.64*	-5.84*	0.05	-3.22*	2.55*	-5.75*
	MSTRG.16648.1	*Glutathione S-transferase theta-1*	H	-5.07*	-4.87*	-2.65*	0.18	2.27*	-2.08*
	MSTRG.10959.1	*Glutathione S-transferase kappa 1-like isoform X1*	H	-1.23*	-1.20*	2.26*	0.01	3.34*	-3.31*
	MSTRG.16599.17	*Glutathione S-transferase 1-like isoform X1*	H	2.13*	2.17*	2.78*	0.02	0.50	-0.47
	MSTRG.11765.2	*Peroxiredoxin-like 2A*	Ha	-0.17	-0.36	5.52*	-0.19	5.68*	-5.89*
	MSTRG.11765.2	*Peroxiredoxin-like 2A*	H	1.00	3.54*	1.12	2.54	-0.05	2.60
	MSTRG.27621.1	*Peroxiredoxin 6*	Ha	-2.03	-0.39	2.48*	1.64	4.52*	-2.88*
	MSTRG.27621.1	*Peroxiredoxin 6*	H	-1.30*	-1.71*	-0.62	-0.42	0.544	-0.95
	MSTRG.16525.8	*Peroxiredoxin*	H	-6.75*	-6.54*	-6.41*	0.19	0.21	-0.01
	MSTRG.251.36	*Thiredoxin reductase*	Ha	3.53*	2.53*	1.97	-1.02	-1.61	0.55
	MSTRG.251.36	*Thiredoxin reductase*	H	3.03*	2.20*	-2.27*	-0.85	-5.43*	4.62*
	MSTRG.5086.22	*Dual oxidase 2*	Ha	-0.21	-0.14	1.48*	0.06	1.69*	-1.64*
	MSTRG.5086.22	*Dual oxidase 2*	H	-3.58	-6.66	-7.25*	-3.10	-3.80	0.74
	MSTRG.19322.3	*Selenoprotein W-like*	Ha	-2.76*	-0.49	-0.26	2.26	2.51	-0.25
	MSTRG.19322.3	*Selenoprotein W-like*	H	-1.43*	-1.98*	-1.25*	-0.57	0.03	-0.60
	MSTRG.20593.2	*Selenoprotein*	Ha	-3.01*	-1.23	0.82	1.77	3.84*	-2.07
	MSTRG.20593.2	*Selenoprotein*	H	-1.72*	-1.94*	-3.08*	-0.24	2.11*	-2.34*
	MSTRG.10820.4	*Selenoprotein M*	H	-6.87*	-2.61	-5.11*	4.25	1.61	2.68
**Chaperones**									
	MSTRG.3389.1	*Heat shock protein 90*	Ha	-1.55	-0.82	4.94*	1.16	6.50*	-5.33*
	MSTRG.3389.1	*Heat shock protein 90*	H	1.30*	1.35*	0.14	-0.03	-1.30*	1.33*
	MSTRG.19712.2	*Heat shock protein 70*	Ha	-2.22	-0.70	4.49*	1.51	6.72*	-5.20*
	MSTRG.19712.2	*Heat shock protein 70*	H	0.30	0.49	0.74*	0.42	1.08*	-0.64*
	MSTRG.19618.1	*Calreticulin*	Ha	-1.29	-0.29	3.23*	0.99	4.54*	-3.54*
	MSTRG.19618.1	*Calreticulin*	H	0.32	-0.69	3.43*	-1.03	2.97*	-3.99*

AB: post-molt; C: inter-molt; D: per-molt; E: ecdysis. ^a^Fold changes (Log_2_ ratio) in gene expression. *Represents a difference between the two molt stages.

In addition, several genes of the antioxidative enzymes system and chaperones also involved in immunity response of *P. trituberculatus*. Thus, for the antioxidative enzymes system, 20 genes were identified in the hemolymph and hepatopancreas, namely, *NADPH oxidase*, *copper/zinc superoxide dismutase 2* (*CuZnSOD-2*), *CuZnSOD-4, ecCuZnSOD, glutathione peroxidase-like isoform X1*, *glutathione peroxidase 7-like*, *glutathione peroxidase*, *glutathione peroxidase 3*, *glutathione S-transferase*, *glutathione S-transferase theta-1*, *glutathione S-transferase kappa 1-like isoform X1*, *glutathione S-transferase 1-like isoform X1*, *peroxiredoxin-like 2A*, *peroxiredoxin*, *peroxiredoxin 6*, *thiredoxin reductase*, *dual oxidase 2*, *selenoprotein*, *selenoprotein W-like*, and *Selenoprotein M*. Moreover, three genes related to chaperones also showed various changes pattern in different molting stages, such as heat shock protein 90 (*HSP90*), *HSP70*, and *calreticulin*.

### Expression patterns of immune-related genes during the molting cycle

**Figure 7:**
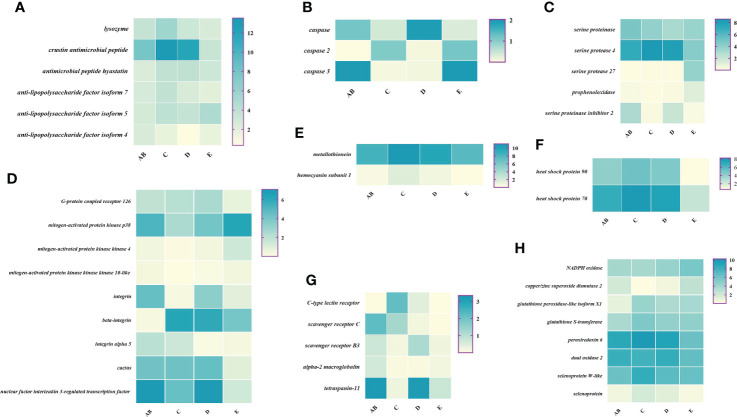
Expression patterns of immune-related genes in the hemolymph during the molting cycle of *P. trituberculatus*. **(A)** The genes related to antimicrobial peptides; **(B)** the genes related to apoptosis; **(C)** the genes related to prophenoloxidase cascade; **(D)** the genes related to signal transduction; **(E)** the genes related to other immune molecules; **(F)** the genes related to chaperones; **(G)** the genes related to pattern recognition proteins/receptors; **(H)** The genes related to antioxidative enzymes system. Notes: (1) The *x*-axis shows the molting stages, whereas the *y*-axis represents the name of genes. (2) The color represents the expression level of the gene; the darker the color, the higher gene expression level. (3) The expression level of immune-related genes was shown as log_2_ transcript per million plus one (TPM + 1).

**Figure 8:**
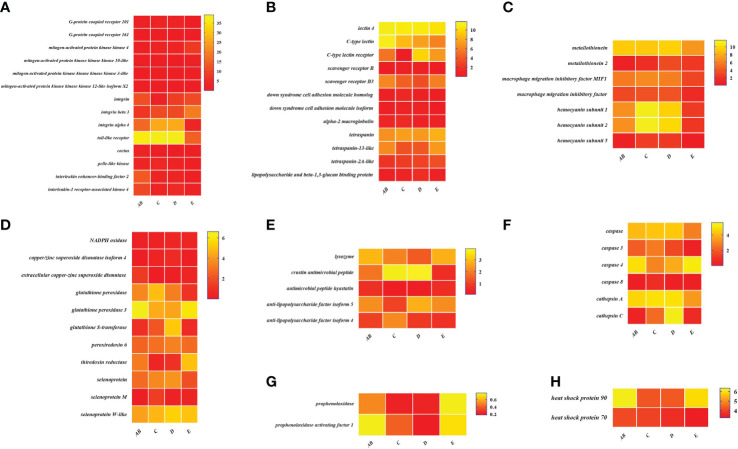
Expression patterns of immune-related genes in the hepatopancreas during the molting cycle of *P. trituberculatus*. **(A)** The genes related to signal transduction; **(B)** the genes related to pattern recognition proteins/receptors; **(C)** the genes related to other immune molecules; **(D)** the genes related to antioxidative enzymes system; **(E)** the genes related to antimicrobial peptides; **(F)** the genes related to apoptosis; **(G)** the genes related to prophenoloxidase cascade; **(H)** the genes related to chaperones. Notes: (1) The *x*-axis shows the molting stages, whereas the *y*-axis represents the name of genes. (2) The color represents the expression level of the gene; the lighter the color, the higher the gene expression level. (3) The expression level of immune-related genes was shown as log2 transcript per million plus one (TPM + 1).

To better understand the changes of immune-related genes in the hemolymph and hepatopancreas during the molting cycle, the expression level of key genes involved in signal transduction, pattern recognition proteins/receptors, apoptosis, prophenoloxidase cascade, antimicrobial peptides, antioxidative enzymes system, chaperones, and other immune molecules were analyzed (**Figures 7**, **8**). The results showed that the expression levels of *p38 MAPK*, *MAPKK4*, *MAPKK3*, *MAPKKK10*, and *MAPKKK12* decline from stage AB to stage C and then rise from stage D to stage E. However, the expression level of *GPCR 126*, *GPCR 161*, and *GPCR 101* rises from stage AB to stage C and then decline from stage D to stage E. Moreover, the higher expression level of *integrin*, *integrin alpha-4*, and *integrin alpha 5* was found in stage AB, whereas the expression level of *beta-integrin* was lower in stages AB during the molting cycle of *P. trituberculatus*. Remarkably, the expression level of *Toll-like receptor* was higher during the molting cycle of *P. trituberculatus* and reached the highest level in stage D. For pattern recognition proteins/receptors, the lowest expression level of 10 genes was found in the E stage, namely, *C-type lectin receptor*, *SRB3*, and *SRC* in the hemolymph and *lectin 4*, *C-type lectin*, *SRB*, *Down syndrome cell adhesion molecule homolog*, *Down syndrome cell adhesion molecule isoform*, and *A2M* in the hepatopancreas. For the other immune molecules, the expression level of those genes reached the highest level in stage C or stage D and the lowest level in E stage or AB stage, such as *metallothionein*, *metallothionein 2*, *macrophage migration inhibitory factor*, *macrophage migration inhibitory factor MIF1*, *hemocyanin subunit 1*, *hemocyanin subunit 2*, and *hemocyanin subunit 5*.

Among the genes related to apoptosis, the expression of *caspase*, *caspase 3*, and *caspase 8* in the hepatopancreas reached the highest level in stage C and the lowest level in stage E. The transcript levels of the genes (*prophenoloxidase*, *prophenoloxidase-activating factor 1*, and *serine protease 27*) that involved in activating proPO system increased significantly from stage D to stage E, whereas the transcript levels of *serine proteinase inhibitor 2* decreased significantly from stage D to stage E. Among the genes related to antimicrobial peptides, the highest expression level of *lysozyme* in hemolymph was found in the E stage while the lowest expression level of *lysozyme* in hepatopancreas was found in the E stage. The expression levels of *crustin* and *antimicrobial peptide hyastatin* in the stage C or stage D significantly higher than AB stage or E stage in the hemolymph and hepatopancreas. The expression levels of *ALF4*, *ALF5*, and *ALF7* in the hemolymph and hepatopancreas showed various changes pattern in different molting stages.

For the antioxidative enzymes system and chaperones, the expression level of *copper/zinc superoxide dismutase isoform 4*, *glutathione peroxidase*, *glutathione S-transferase*, *peroxiredoxin*, *peroxiredoxin 6*, and *dual oxidase 2* in stage C or stage D significantly higher than that of stage E or stage AB. Moreover, the expression level of *selenoprotein* (*selenoprotein M* and *selenoprotein W-like*) increased significantly from stage AB to stage C and then decline gradually from stage C to stage E. The expression level of chaperones (*HSP90* and *HSP70*) gradually decreased from stage C to stage E.

### Validation of gene expression by quantitative real-time PCR

**Figure 9:**
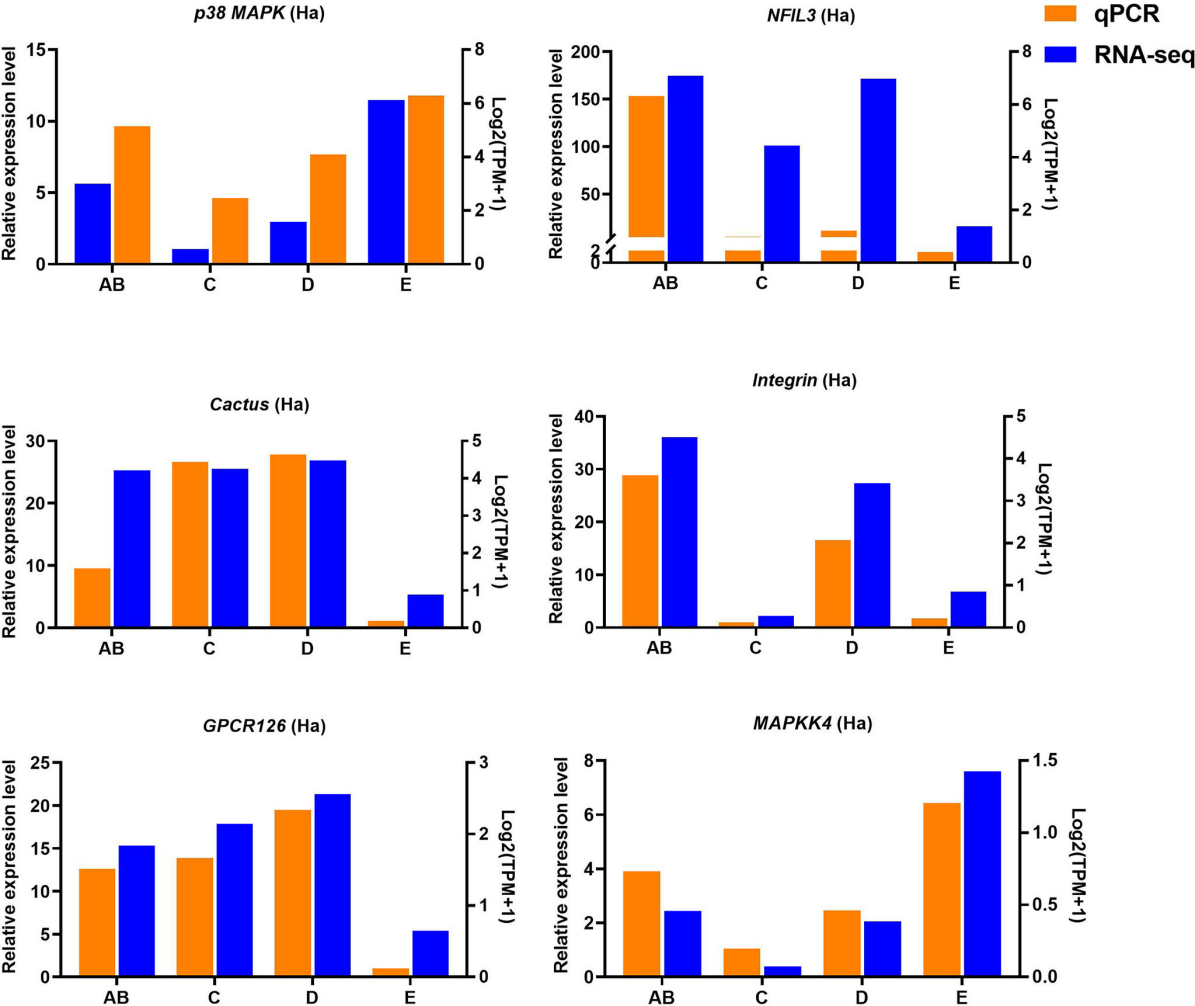
Quantitative expression of six genes was determined by qPCR in hemolymph from different molting stage of *P. trituberculatus*. p38 MAPK: *p38 mitogen-activated protein kinase*; NFIL3: *nuclear factor interleukin 3–regulated transcription factor*; GPCR126: *G-protein coupled receptor 126*; *MAPKK4*: *mitogen-activated protein kinase kinase 4*.

**Figure 10:**
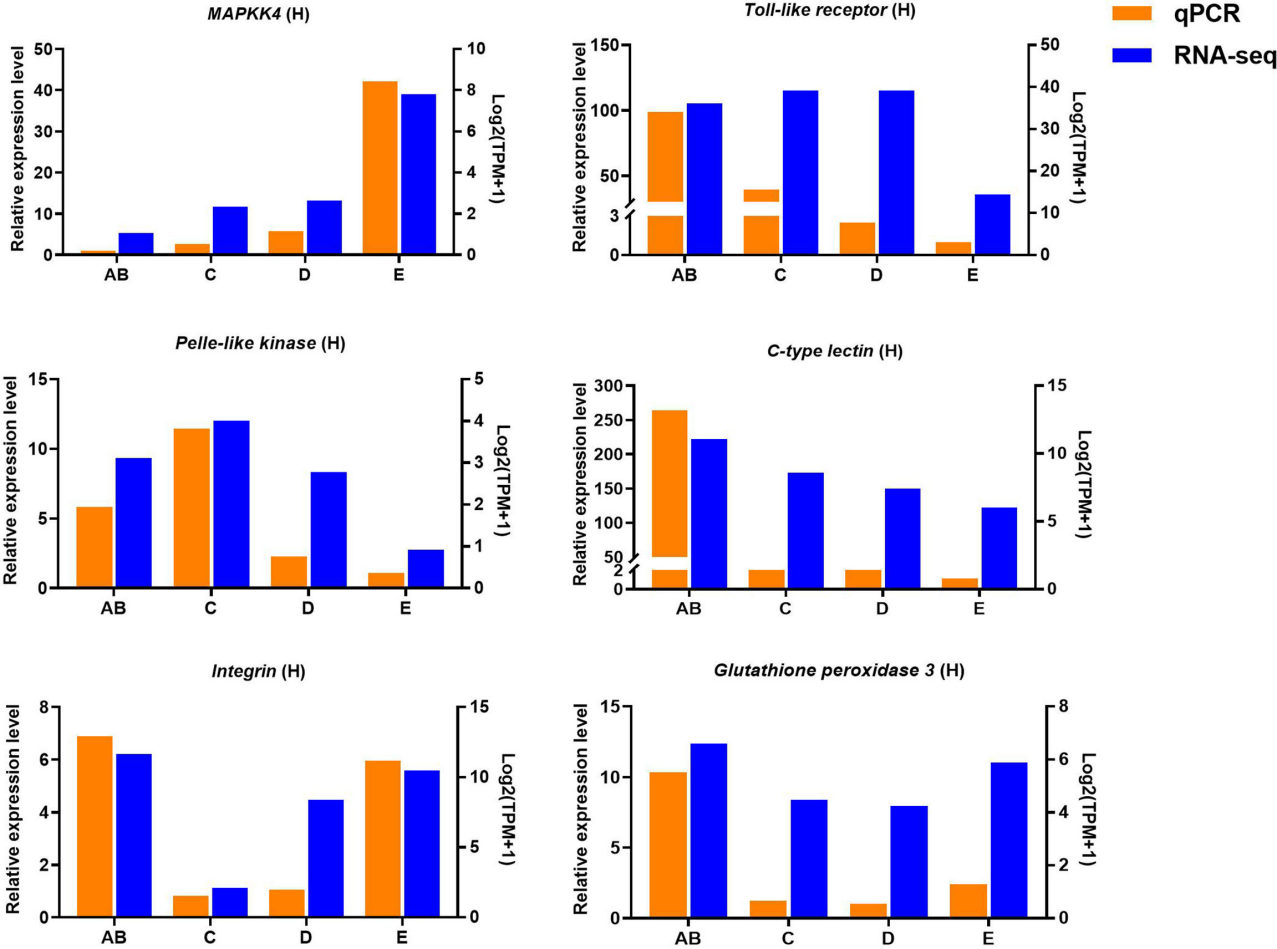
Quantitative expression of six genes was determined by qPCR in hepatopancreas from different molting stage of *P. trituberculatus*. *MAPKK4: mitogen-activated protein kinase kinase 4*.

Ten selected important genes in the molting cycle of P. trituberculatus were determined using qRT-PCR to validate the RNA-seq results. The qPCR results showed that the relative expression patterns of these nine genes were consistent with the RNA-seq results (**Figures 9**, **10**). Notably, the qRT-PCR results showed that the expression level of *Toll-like receptor* in the hepatopancreas of stages AB to D was higher than that of stage E, and reached the highest level in stage AB. However, the RNA-seq results showed that the expression level of *Toll-like receptor* in the hepatopancreas of stages AB to D was higher than that of stage E, and reached the highest level in stage D.

## Discussion

Like other invertebrates, crustaceans rely entirely on the innate immune system to combat invading pathogenic microorganisms, which mainly consists of cellular and humoral immunity (26). Specifically, the cellular immunity involves phagocytosis, apoptosis, nodulation, and encapsulation, whereas the humoral immunity includes antimicrobial peptides (AMPs), prophenoloxidase cascade, clottable proteins, the toll and immune deficiency (IMD) pathway, and so forth (27). Previous studies have shown that the crustacean immune system is a tightly regulated network, and the immune molecules that synthesized or released by hemolymph and hepatopancreas are able to maintain the balance of immune homeostasis under normal physiological conditions (27, 28). As an effective physical barrier, the hard carapace and external cuticle are the crab’s first line of immune defense, protecting the crab from the attachment and penetration of pathogens (28). However, the crabs are too vulnerable to pathogenic bacteria when they are molting or just finished molting. Thus, this study performed a comprehensive transcriptome analysis of the hemolymph and hepatopancreas in different molting stage of *P. trituberculatus*, aiming to uncover the immune-related to genes or pathway altered during the four molting stages and further improve the understanding of the immune mechanisms applicable to the molting cycle process in crustaceans.

In this study, the significantly changed molecules associated with immune processes during the molting cycle of *P. trituberculatus* could divided into seven functional categories, namely, antimicrobial peptides (AMPs), blood clotting system, prophenoloxidase (proPO) cascade, pattern recognition receptors (PRRs), signal transduction, apoptosis, and other immune molecules (**Table 5**). AMP are key components of innate immunity and play key roles in the defense against invading microorganisms, including lysozyme, crustin, and so forth (29). Lysozymes can be classified into six types, and the c-type lysozymes have been reported in shrimp, crabs, and crayfish (30). Pan et al. (31) reported that the c-type lysozymes of *P. trituberculatus* may be an inducible acute-phase protein and bacterial challenge could decline its mRNA expression (31). Crustin group, the largest family of crustacean AMPs, exhibits broad antimicrobial activity against Gram-negative bacteria (32). Anti-lipopolysaccharide factors (ALFs) are considered to have broad-spectrum antimicrobial activity against Gram-positive bacteria, Gram-negative bacteria, fungi, and viruses (33). The present study showed that the highest expression level of several AMPs (*crustin*, *lysozyme*, *antimicrobial peptide hyastatin*, and *anti-lipopolysaccharide factor isoform 7*) was found in the stage C hemolymph, indicating that the higher antimicrobial activity was found in this stage. Moreover, the lowest expression level of *crustin, antimicrobial peptide hyastatin*, and *anti-lipopolysaccharide factor isoform 4/5/7* was found in the stage E, which may lead to the lower antimicrobial activity of crab in this stage and susceptible to bacterial or virus infection.

The prophenoloxidase (proPO) cascade system is one of the most essential immune reactions, which involves the participation of proPO, phenoloxidase (PO), serine proteases, and inhibitors (34). The Phenoloxidase (PO) is believed to be derived from its precursor prophenoloxidase (proPO), which is considered as the rate-limiting step of melanin formation (35, 36). Melanization is an important component of the innate immune responses in arthropods and allowing a rapid response to pathogen infection (36). Serine proteases, especially those with one or two clip domains, play critical roles in the activation of prophenoloxidase (proPO) activating system (37). Serine proteinase inhibitors have been functionally characterized as negative regulators of the proPO activation cascade by inhibiting the serine proteases in different crustacean species (35, 38). In the present study, the transcript levels of the genes (*prophenoloxidase*, *prophenoloxidase-activating factor 1*, and *serine protease 27*) that involved in activating proPO system increased significantly from stage D to stage E, suggesting that the proPO cascade system is involved in the immune defense of *P. trituberculatus* during ecdysis. The transcript levels of *serine proteinase inhibitor 2* that inhibiting in activating proPO system decreased significantly from stage D to stage E further confirmed the above speculation.

Pattern recognition receptors (PRPs) are a group of germ line encoded receptors that recognize common epitopes on pathogen surfaces and then trigger a series of downstream responses (39). In this study, a series of PRRs were identified, namely, *lectin 4*, *C-type lectin*, *C-type lectin receptor*, *scavenger receptor B* (*S*RB), *SRC*, *Down syndrome cell adhesion molecule homolog*, alpha-2 macroglobulin, *lipopolysaccharide and beta-1,3-glucan binding protein*, and *tetraspanin*, indicating that the immune defense mechanism of crab has a very complex recognition network. Lectins are carbohydrate-binding proteins that can be classified into several families and C-type lectins families are the largest and most distributed in crustaceans (40). Previous studies reported that the C-type lectins not only can regulate the expression of antilipopolysaccharide factor and inhibit bacterial growth but also participate in phagocytosis, encapsulation, and the activation and regulation of the proPO system (41–44). The present study showed that the expression level of *C-type lectins* and *lectin 4* higher than that of other PRPs, demonstrating that the *lectins* play a key role in recognizing invading microorganisms during the molting cycle of *P. trituberculatus*. Moreover, the results showed that the expression level of *C-type lectins* and *lectin 4* in the hepatopancreas decreased continuously from stage AB to stage E, which is similar to the result of *Scylla paramamosain* (11). However, the expression level of *C-type lectin receptor* in the hepatopancreas dramatically increased from stage C to stage E, illustrating that C-type lectin receptor played a key role in trigger other intracellular signal pathways. Moreover, Sanchez-Salgado et al. (45) reported that the receptor of lectin was β-integrin when the lectin involved in the phagocytosis of shrimp *Fenneropenaeus chinensis* (45). SR comprised nine heterogeneous classes, of which SRB and SRC promote phagocytosis of viruses in crustaceans (46, 47). The transcript of *SRB* and *SRC* in the hemolymph and hepatopancreas showed that the opposite expression pattern from the stage AB to stage C may suggest that the different role of two tissues in SR mediates phagocytosis. However, the lowest expression level of *SRB* and *SRC* in the hemolymph and hepatopancreas was found in stage E, indicating that the low phagocytosis in this stage.

As an immune effector, Down syndrome cell adhesion molecule (*Dscam*) can produce thousands of isoforms and specifically binds to different types of bacteria and promotes challenge-specific protection (48, 49). Lipopolysaccharide-and beta-1,3-glucan-binding protein (LGBP) plays a crucial role in immune mechanism by mediating the recognition of invading pathogens to host cells (50). In this study, the expression level of hepatopancreatic *Dscam* and *LGBP* in stage C/D significantly higher than that in stage AB/E, demonstrating that the crab in stages C and D are able to make a rapid immune response when invaded by pathogens. Alpha-2-macroglobulin (A2M) is highly upregulated upon microbial infection and involved in several immune pathways (51). The transcript of *alpha-2 macroglobulin* in the hemolymph and hepatopancreas showed that a decline trend from the stage AB to stage E may suggest that the A2M was not necessarily to participate in the immune defense during the molting cycle of crab. Tetraspanins, belong to the transmembrane 4 superfamily (TM4SF), are highly involved in viral and bacterial infections (52, 53). Previous studies showed that the tetraspanin-3 and tetraspanin 8 play a key role in *WSSV* and *Aeromonas hydrophila* infection of shrimp (52, 53). Li et al. (54) reported that *tetraspanin-18* may play an important role in the beginning of molting cycle of *Eriocheir sinensis* (54). The present study showed that tetraspanin, tetraspanin-13-like and tetraspanin-2A-like were identified, and its higher transcript level was detected in the hepatopancreas during the molting cycle of *P. trituberculatus*. It was speculated that the tetraspanins may involve in the immune defense or other physiological process of *P. trituberculatus.*

G-protein coupled receptors (GPCRs) are important transmembrane receptors that played a vital role in the innate immune defense of crustacean, including the defense pathogens, melanization, sclerotization, and bacterial infection (55–57). Mitogen-activated protein kinase (MAPK) signaling cascade consists of extracellular signal-regulated kinases (ERKs), C-Jun N-terminal or stress-activated protein kinases (JNKs/SAPKs), and p38 MAPKs pathways, which and are involved in cellular response to inflammatory cytokines, environmental stress, and pathogenic infection (58, 59). Among them, p38 MAPK can be activated by various extracellular stimuli and play a critical role in innate immune responses by induces the expression of *interleukin-1* and *tumor necrosis factor-alpha* and initiates (60, 61). Mitogen-activated protein kinase 4 is a key upstream kinase in the JNK/p38 MAPK pathway that has been reported to play an anti-bacterial role in shrimp by regulate the expression of antimicrobial peptides (62, 63). In this study, the expression level of *GPCR 126*, *GPCR 161*, and *GPCR 101* rise from stage AB to stage C and then decline from stage D to stage E. However, the expression level of *p38 MAPK*, *MAPKK4 MAPKKK3*, *MAPKKK10*, and *MAPKKK12* decline from stage AB to stage C and then rise from stage D to stage E. The opposite expression pattern of *GPCR* and *MAPK* family genes showed that the GPCR signaling pathway and MAPK signaling cascade play a critical role in the innate immune responses of stage C/D or stage AB/E, respectively.

Integrins belong to a large family of heterodimeric cell surface receptors that composed of α and β subunits (64). Previous studies have shown that integrin involved in WSSV infection, bacterial infection, proPO activation, and phagocytosis of shrimp and crab (65–69). The present study showed that the higher expression level of *integrin*, *integrin alpha-4*, and *integrin alpha 5* was found in stage AB, illustrating that integrin-mediated signal transduction has significant function in balanced immune defense of this stage. Moreover, Lin et al. (2013) reported that the expression level of *integrin beta* in the haemocytes was lower in stages D and higher in stages AB during the molting cycle of white shrimp *Litopenaeus vannamei* (66). In this study, the expression level of *beta*-*integrin* in the haemocytes was lower in stages AB and higher in stages C/D during the molting cycle of *P. trituberculatus*. The opposite expression pattern of *beta*-*integrin* in two animals may be related to species specificity.

Toll-like receptor (TLR) signaling plays an essential role in initiating innate immune responses in crustaceans (70). Specifically, the activation of the Toll pathway is initiated by the Spätzle and then Spätzle binds the N terminus of Toll inducing a conformational change. Subsequently, the trimeric complex MyD88/Tube/TRAF6 is formed then transducing signals to the Dorsal/Cactus complex that regulates the Toll-dependent gene expression of AMPs, and a significant number of innate immune responsive genes (71). In this study, four significantly expressed genes (*Toll-like receptor*, *cactus*, *pelle-like kinase*, and *nuclear factor interleukin 3-regulated transcription factor*) involve in TLR signaling were identified and the higher expression level of *Toll-like receptor* was found from stage AB to stage D of *P. trituberculatus*, further demonstrating that the TLR signalling pathway play an important role of innate immunity of this crab. Notably, the qRT-PCR results showed that the expression level of *Toll-like receptor* in the hepatopancreas reached the highest level in stage AB, whereas the RNA-seq results showed that the expression level of *Toll-like receptor* in the hepatopancreas reached the highest level in stage D. We speculate the possible reasons for this difference are the differences in detection methods or samples.

Apoptosis is a fundamental cellular process that plays a critical role in innate immunity, normal development, and tissue homeostasis (72, 73). Caspases are a conserved family of cysteine proteases, which could initiate and execute apoptosis, and the apoptotic caspases are further categorized into initiator caspases (caspase-2, -8, -9, and -10), inflammatory caspase (caspase-1, -4, and -5) and effector caspases (caspase-3, -6, and -7) (74, 75). In crustacean, increasing evidences have indicated that caspase -2, -3, and -8 could suppress WSSV infection *via* apoptosis induction (76–78). The present study showed that the expression level of caspases (caspase-2, -3, -4, and -8), changed significantly during the molting cycle of *P. trituberculatus*, indicating that caspase would contribute to apoptosis homeostasis and immune defense of this crab. Hemocyanin, the respiratory protein of arthropods and mollusks, has been linked to key aspects of innate immunity, in particular antibacterial, antiviral and induced the phenoloxidase-like activities (79). The expression level of hemocyanin subunit (1, 2, and 5) in this study increased significantly from stage AB to stage C and then decline gradually from stage C to stage E, illustrating that the activity of hemocyanin-mediated immune defense was highest in the stage C and lowest in the stage E.

During pathogen invasion, innate immunity can produce free radical molecules and their derivatives reactive nitrogen/oxygen species (RNS/ROS) against invading pathogens in shrimps and crabs (80, 81). Previous study has established that the NADPH oxidase play important roles in host defense system by catalyzing the production of superoxide anions (82). In this study, the expression level of *NADPH oxidase* increased gradually from stage AB to stage E in the hemolymph and hepatopancreas, suggesting that crabs may carry out the immune defense at this stage by increasing the content of superoxide anion to protect themselves. Although ROS are beneficial for host defense against pathogens, their accumulation damages cells and disrupts immune system homeostasis (83). Thus, organisms eliminate excessive ROS through the antioxidant systems composed of some endogenous antioxidant enzymes, such as superoxide dismutase (SOD), glutathione peroxidase (GPx), glutathione S-transferase (GST), and peroxiredoxin (Prx) (84, 85). It has been reported that those antioxidant enzymes can collaboratively contribute to the homeostasis of redox status in organisms, and those enzymes were also closely involved in the crustacean immune responses (81, 86–88). Specifically, the mRNA expressions of *copper/zinc superoxide dismutase* (*Cu/ZnSOD*), *extracellular copper-zinc superoxide dismutase* (*ecCu/ZnSOD*) and *glutathione-s-transferases* (*GSTs*) significantly upregulated when the crustacean challenging with virus infection (89–91). Mu et al. (86) reported that the involvement of *peroxiredoxins 6* in responses against bacterial infection (86). In this study, the transcript of three SOD isoforms showed opposite expression trends during the molting cycle of *P. trituberculatus*, indicating that SOD can protect the body from injury by maintaining ROS homeostasis in crabs. Moreover, the transcript of *glutathione peroxidase*, *glutathione-s-transferases* (*GSTs*) and *peroxiredoxin 6* significantly increased from stage AB to stage D and then decreased in stage E, illustrating that those endogenous antioxidant enzymes are important in maintaining redox equilibrium in crab.

As an antioxidant, selenoprotein M and selenoprotein W-like have been reported that involved in alleviating the oxidative stress and apoptosis caused by virus infection (92, 93). The expression level of *selenoprotein* (*selenoprotein M* and *selenoprotein W-like*) in this study increased significantly from stage AB to stage C and then decline gradually from stage C to stage E, indicating that the alleviation effect of selenoprotein on oxidative stress was the highest in the C stage and the lowest in the E stage. Heat shock proteins (HSPs), also known as stress proteins and extrinsic chaperones, are essential for the maintenance of cellular proteostasis (94). In recent years, increasing evidences have reported that HSPs (particularly HSP70) elicit a variety of immune responses to infection and modulate several inflammatory cascades (95–97). The higher expression level of *heat shock protein 70* in this study was found from stage AB to stage C and then significantly decline in stage E, indicating that HSP 70 cannot effectively activate the immune pathway in stage E. Moreover, the transcript of HSP 90 in the hemolymph and hepatopancreas showed opposite expression pattern during the molting cycle of *P. trituberculatus* may relate to the multiple roles of HSP90. It has been reported that the HSP90 participated in the process of lipid droplet biogenesis and lipids homeostasis (98, 99). Except for the immune function, the hepatopancreas of crustaceans is the main organ for lipid metabolism, which provides energy for growth and development (100, 101).

## Conclusions

**Figure 11:**
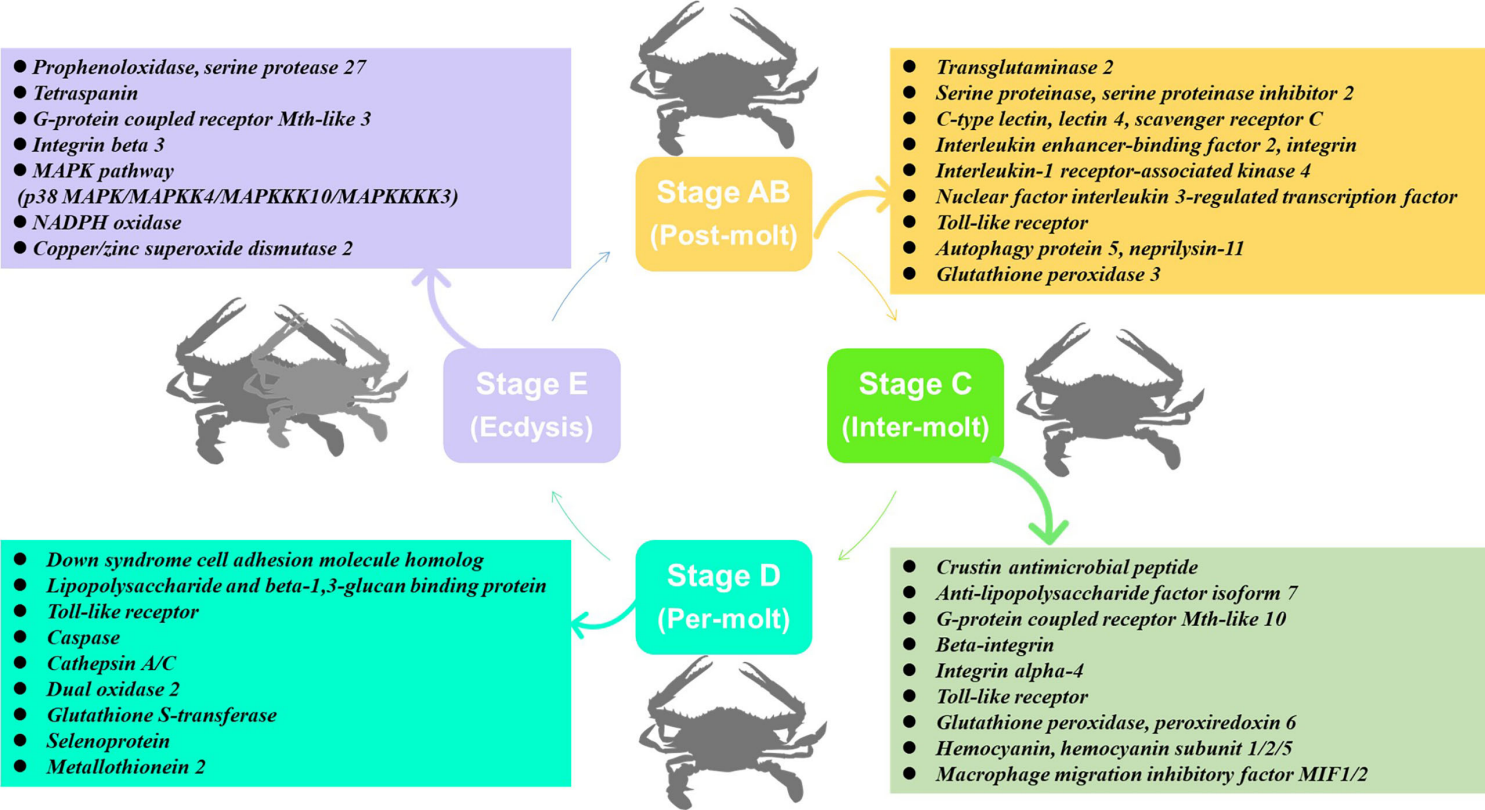
The speculated key genes and pathway for immune response during the molting cycle of *P. trituberculatus* based on the transcriptome analysis.

In this study, transcriptome sequencing has explored the expression level of immune-related genes in the hemolymph and hepatopancreas during the molting cycle of *P. trituberculatus*. The present results showed that the expression levels of immune-related genes changed significantly throughout the molting cycle of *P. trituberculatus*. For example, genes related to GPCR and MAPK signalling pathways exhibit opposite expression patterns from stage AB to stage E. The expression level of *Toll-like receptor* was higher from stage AB to stage D of *P. trituberculatus.* The lowest expression level of ten genes related to pattern recognition proteins/receptors in the hepatopancreas was found in the E stage. The transcript levels of the genes (*prophenoloxidase*, *prophenoloxidase-activating factor 1*, *serine protease 27*) that involved in activating proPO system increased significantly from stage D to stage E. Moreover, the expression level of *copper/zinc superoxide dismutase isoform 4, glutathione peroxidase*, *glutathione S-transferase*, *peroxiredoxin*, *peroxiredoxin 6*, and *dual oxidase 2* in stage C or stage D significantly higher than that of stage E or stage AB. The speculated keys genes and pathway for immune response during the molting cycle of *P. trituberculatus* based on the transcriptome analysis is summarized in **Figure 11**. This study results not only provide basic information to discover genes and pathways involved in immune regulation on the molting of swimming crab but also provided important clues for the innovation of crustacean disease prevention and control technology.

## Data availability statement

The data presented in the study are deposited in the Sequence Read Archive (SRA) repository, accession number: PRJNA855992.

## Author contributions

ML: Experimental design, Writing - Original Draft, Data Curation. HN: Formal analysis, Data Curation. XZ: Data Curation, Validation. QS: Data Curation, Visualization. XW: Experimental design, Supervision. JH: Writing-Editing, Funding acquisition.

## Funding

This study was funded by the two projects (No. 42106088 and No. 32172993) from the Natural Science Foundation of China, an open fund from Zhejiang Marine Fisheries Research Institute (2021KF005), a Basic Public Welfare Program of Zhejiang Province (Grant No. LGN22C190005), a Project of Major Agricultural Technology Cooperation Plan of Zhejiang Province (Grant No. 2020XTTGSC03 and No. 2022XTTGSC04), an Open-end Funds (SH20201205) of Jiangsu Key Laboratory of Marine Bioresources and Environment, a practice innovation training program projects (No. 202111641127Y and No. SY202257X) for the Jiangsu College students. Infrastructure costs were partially supported by the Project of Jiangsu Fisheries Science 537 and Technology (SZ-LYG202029).

## Conflict of interest

The authors declare that the research was conducted in the absence of any commercial or financial relationships that could be construed as a potential conflict of interest.

## Publisher’s note

All claims expressed in this article are solely those of the authors and do not necessarily represent those of their affiliated organizations, or those of the publisher, the editors and the reviewers. Any product that may be evaluated in this article, or claim that may be made by its manufacturer, is not guaranteed or endorsed by the publisher.
